# Fundamentals of *in Situ* Digital Camera Methodology for Water Quality Monitoring of Coast and Ocean

**DOI:** 10.3390/s90705825

**Published:** 2009-07-22

**Authors:** Lonneke Goddijn-Murphy, Damien Dailloux, Martin White, Dave Bowers

**Affiliations:** 1 Department of Earth and Ocean Sciences, National University of Ireland, Galway, Ireland; E-Mail: martin.white@nuigalway.ie (M.W.); 2 Current address: Environmental Research Institute, North Highland College, UHI Millennium Institute, Thurso, Caithness, Scotland, KW14 7JD, UK; 3 Laboratoire des Sciences, Appliques au Génie Civile et Côtier, Université de Pau et des Pays de l’Adour, 1 allée du parc Montary, 64600 Anglet, France; E-Mail: damien.dailloux@univ-pau.fr; 4 AZTI-Tecnalia, Herrera Kaia, Portu aldea z/g, 20110 Pasai (Gipuzcoa), Spain; 5 School of Ocean Sciences, University of Wales, Menai Bridge, Gwynedd LL59 5EY, UK; E-Mail: oss063@bangor.ac.uk

**Keywords:** digital, camera, ocean, colour, marine, technology, advancement

## Abstract

Conventional digital cameras, the Nikon Coolpix885^®^ and the SeaLife ECOshot^®^, were used as *in situ* optical instruments for water quality monitoring. Measured response spectra showed that these digital cameras are basically three-band radiometers. The response values in the red, green and blue bands, quantified by *RGB* values of digital images of the water surface, were comparable to measurements of irradiance levels at red, green and cyan/blue wavelengths of water leaving light. Different systems were deployed to capture upwelling light from below the surface, while eliminating direct surface reflection. Relationships between *RGB* ratios of water surface images, and water quality parameters were found to be consistent with previous measurements using more traditional narrow-band radiometers. This current paper focuses on the method that was used to acquire digital images, derive *RGB* values and relate measurements to water quality parameters. Field measurements were obtained in Galway Bay, Ireland, and in the Southern Rockall Trough in the North Atlantic, where both yellow substance and chlorophyll concentrations were successfully assessed using the digital camera method.

## 1. Introduction

Remote sensing offers a particularly useful way to monitor water resources over different space and time scales. Satellite spatial resolution, on the order of 1 km, while useful in shelf seas, cannot be used in coastal areas and inland waters where shallow water and land borders contaminate the images. In such regions, *in situ* or airborne mounted systems must be employed. At present, narrow-band, multi-spectral radiometers have been used to measure optical properties of natural waters to yield information on many water quality parameters, such as chlorophyll [1–7], mineral suspended sediments (MSS) [8–10] and yellow substance [11–13]. The use of relatively complex multi-spectral instruments may be costly, however. This current study explores the possibilities of employing a conventional digital camera, as an alternative low-cost technique, to estimate water composition from optical properties of the water surface.

The concept of using a conventional imaging camera as a remote sensing instrument is not unknown. The Kodak DCS460c^®^ digital camera, a camera designed for photojournalism, has been used for area mapping of vegetation [14], and urban areas [15]. Directional reflectance of grass and of pine trees was studied using a standard CCD camera, an Electrim EDC-1000C^®^ [16]. The benefits of aerial imaging photography have also been applied to hydrographical research. A setup of four synchronous analogue K-17 cameras and filters mounted on an airplane was applied to measure the roughness of the sea surface from photographs of the sun’s glitter [17]. Frontal systems in a coastal zone were studied using an airborne standard 35 mm camera [18]. Ocean surface currents were accurately estimated using a digital camera system on an aircraft, by deriving the Doppler shift of gravity waves from the images [19]. An airborne Kodak DCS460c was applied in studies of the dynamics of a river plume, discharging into coastal water [20]. Cameras have been used *in situ* as well, an example is the oceanographic camera system designed to measure underwater radiance distribution in natural waters [21]. The system contained two analogue Nikon cameras placed back-to-back, each with a fisheye lens and could operate at depths up to 100 m. In more recent times, an *in situ* Sony Mavica FD 83^®^ digital camera, with a pipe breaking the water surface attached to its lens, was used to estimate MSS levels in coastal waters [20]. Operating a Nikon Coolpix 885 digital camera in a similar fashion, yellow substance and chlorophyll concentrations were successfully monitored in Galway Bay, Ireland [22].

Employing an off the shelf digital camera as an *in situ* optical instrument to assess water composition is a relatively novel technique [20,22]. The method described in those papers is the focus of this paper. We explored remote sensing algorithms known to calculate yellow substance and chlorophyll concentrations, as these are the optically active components that control the water colour in Galway Bay [22]. The remote sensing algorithms we applied were based on observations of subsurface spectral reflectance *R(λ_i_*), defined by the ratio of up welling (ir)radiance and down welling (ir)radiance. Yellow substance absorption decreases exponentially with increasing wavelength from ultraviolet to near infrared wavelengths, giving water a yellow colour [23]. Other names for yellow substance are: coloured dissolved organic matter or CDOM, aquatic or marine humus, gilvin and gelbstoff. To quantify the concentration of yellow substance, the absorption coefficient at 440 nm, ah(440), is commonly used [23]. When yellow substance and water are the main light absorbers, the red/blue reflectance ratio will linearly increase with yellow substance concentration [11–13]:

(1) $$\mathrm{ah}(440)=n_{\mathrm{ys}}\;R\left(\lambda_{r}\right)/R\left(\lambda_{b}\right)+m_{\mathrm{ys}}$$

Known remote sensing algorithms for assessing chlorophyll concentration, [Chl], are described by a log-log relationship with the blue/green reflectance ratio [1,2,4,5,24]:

(2) $$[\mathrm{Chl}]=m_{c}{\left(\frac{R\left(\lambda_{b}\right)}{R\left(\lambda_{g}\right)}\right)}^{n_{c}}\text{or}\;\text{ln}([\mathrm{Chl}])=n_{c}\;\text{ln}\left(\frac{R\left(\lambda_{b}\right)}{R\left(\lambda_{g}\right)}\right)+\text{ln}\left(m_{c}\right)$$

Chlorophyll absorption is low in the green, but high in the blue part of the spectrum, and hence the blue/green colour ratio of reflectance decreases with increasing chlorophyll concentration, if chlorophyll dominates the water colour.

The outline of this paper is as follows. First it is demonstrated how images of water leaving light were acquired using different set-ups, and how their *RGB* values were derived. In the following sections is described how the response spectra of the ECOshot and CP885 digital cameras were measured, and how the Ocean Colour Sensor, an *in situ* irradiance meter designed for ocean colour observations, was used. Next, the required lighting conditions for using the *in situ* camera are explained. In the final Methods section, details about the surveys in Galway Bay, Ireland, and in the Southern Rockall Trough in the North Atlantic, can be found. The response spectra of the two cameras are presented in Results. These spectra show that the digital cameras are fundamentally three-band radiometers. The observed relationships between *RGB* ratios and water quality parameters, obtained using the *in situ* CP885 during numerous surveys, are described in the following section. In the last Results section, the findings of the *in situ* CP885 and ECOshot are compared. The digital camera methodology and its results are evaluated in the Discussion, which includes a concise conclusion.

## 2. Methods

### 2.1. Image Acquisition

**Figure 1.**
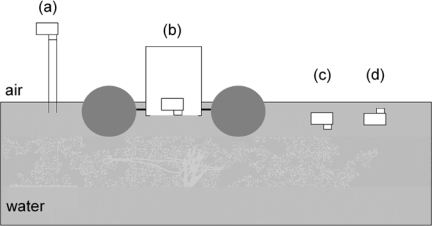
Diagram illustrating camera deployment methods: (a) CP885 with a tube breaking the water surface attached to its lens (b) CP885 in an underwater housing, mounted in a frame kept afloat by four buoys (two shown in diagram) (c) ECOshot underwater camera imaging subsurface up welling light (d) ECOshot underwater camera imaging subsurface down welling light.

Digital pictures of water leaving light were taken in a number of different ways (Figure 1). At all sample stations, the CP885 was used with a plastic tube fitted around the lens [22]. The tube was positioned to break the water surface to prevent surface reflected light from entering the camera, and hence only water leaving radiance was measured (Figure 1a). The bottom end of the tube was held as close to 10 cm depth as possible. Additional pictures were taken, using an underwater camera housing in a floating frame. The CP885 was mounted in the housing, looking down through an optical lens located approximately 10 cm below the waterline (Figure 1b). The system was deployed from the vessel, and let drift about 20 metres away before taking pictures. The camera was operated with the Harbortronics DigiSnap 2000^®^ time-lapse control, its settings configured with the terminal emulator program HyperTerminal on a PC. The SeaLife ECOshot is a waterproof digital camera, designed to take underwater pictures. The ECOshot was held and operated below the water surface, taking pictures of sub surface up welling light, and then inverted to capture down welling sunlight transmitted through the water surface (Figure 1c,d).

Remote sensing algorithms for water composition monitoring are generally based on spectral ratios (Equations 1 and 2), it was therefore decided to use ratios of the camera’s *RGB* values. Since *RGB* ratios appeared to vary significantly with exposure, five pictures were taken in auto exposure mode, at a range of exposure compensation settings, and the *RGB* values were derived where their total, *I_tot_* = *R* + *G* + *B*, was 400 [22]. *R/B* and *B/G* values showed a respective exponential increase and decrease with tube depth, but the tube depth, and its variation, during measurements was found to be negligible in waters as clear as in Galway Bay. For reasons of convenience, only one single picture was taken while imaging subsurface down welling light, using the ECOshot in zero exposure compensation setting.

A digital camera can adjust colours according to the colour of the lighting environment so those colours that appear white to the human eye also appear white when viewed in the final picture. This adjustment is called “white balance” [25]. A changed white balance setting changed the observed colour ratios, it was therefore essential to keep the digital camera in a fixed white balance setting. We used our cameras in the manual white balance setting ‘Direct Sunlight’.

### 2.2. Response Spectra of the Cameras

The response spectra of the CP885 and the ECOshot were measured in a double beam spectrophotometer, the UV-1601^®^. The blazed holographic grating and the deuterium and tungsten-halogen lamps of the UV-1601 produced two beams of light with a range of wavelengths across the UV-VIS spectrum and into the NIR from 190 to 1,100 nm. Spectral data was obtained at constant band pass with resolution of less than 2 nm. Both cameras fitted in the UV-1601 with the lid closed, if the cells and cell holders were taken out of the instrument. Pictures were taken of one of the beams projected onto white paper using the timer control. The wavelengths ranged from 400 to 700 nm, and every 10 nm a picture was taken with the camera set with ‘Direct Sunlight’ white balance. From each picture, the *RGB* values of the visible image of the projected beam were derived, quantifying the response of the three bands to light of a certain wavelength. An experiment using the CP885, in which a sheet of uniform blue colour containing different sized black areas was imaged, showed that the *RGB* values of the blue section didn’t change with the size of the black fraction. It was therefore assumed that the effects of white balancing were similar for the camera used *in situ*, and for the spectrophotometer measurements and that the same applied to the ECOshot. Different exposure compensation values didn’t relate to different response values and one picture per wavelength was taken using zero exposure compensation value, 0 eV. For the CP885, this was achieved by using a 1 s exposure time, and aperture setting f/2.8.

### 2.3. In Situ Measurements Using the Ocean Colour Sensor

During four Galway Bay surveys the Bangor Ocean Colour Sensor (OCS), an instrument designed for ocean colour measurements, was applied to measure subsurface up welling and above surface down welling sunlight. The OCS is a cosine irradiance collector in a waterproof casing, contained in a floating frame similar to Figure 1b. The red, green, cyan and blue channels of the OCS measure irradiance center around wavelengths 668.1, 569.1, 490.1 and 439.6 nm respectively (respective half bandwidths are 11.7, 11.0, 9.3 and 8.9 nm) [7]. At each station the OCS was deployed and let drift a couple of meters away from the vessel. The OCS was positioned faced down to measure up welling light for 5 minutes, then faced up to record down welling light for five minutes followed by five minutes faced down again. The instrument was set to execute continuous 10 second integrations.

Light levels were measured in digital counts (DC). For deriving accurate irradiance values in J s^−1^ m^−2^, the OCS should have been calibrated regularly due to a possible drift of the instrument, preferably before or after each survey. The raw digital counts could be used to measure spectral reflectance correctly, however.

### 2.4. The Colour of Underwater Down Welling Daylight

We estimated reflectance ratios (Equations 1 and 2) by measuring colour ratios of upwelling light, and hence we needed those conditions for which the colour ratios of downwelling light approximated one. *RGB* values of the water surface images were believed to be proportional to water leaving irradiance, *E_wu_(λ_i_*), with *i*, *j* = *R*, *G*, *B* and *λ_i_* the centre wavelength of band *i*. Using *RGB* ratios to approximate spectral ratios of reflectance, the colour ratios of subsurface down welling sunlight, *E_wd_(λ_i_*), were therefore not taken into account:

(3) $$i/j\propto E_{\mathrm{wu}}\;\left(\lambda_{i}\right)/E_{\mathrm{wu}}\;\left(\lambda_{j}\right)=E_{\mathrm{wd}}\;\left(\lambda_{i}\right)/E_{\mathrm{wd}}\;\left(\lambda_{j}\right)\times R\left(\lambda_{i}\right)/R\left(\lambda_{j}\right)$$

Natural daylight over the sea is almost white [26], and hence its colour ratios approximate one. The spectral distribution varies little with changing sky conditions, shifting slightly towards blue wavelengths in the presence of rain and clouds [26]. Measurements while dusk or dawn radiation colour the sky red should be avoided.

Certain circumstances can change the colour of down welling light as it passes through the water surface. Although transmittance of sunlight through the water surface is not dependent on wavelength in itself [27], it is dependent on the fraction of diffuse sunlight, *F*, and *F* can be a function of wavelength [28]. Under a completely overcast sky, *F* equals one, and transmittance is the same for all wavelengths. In the presence of direct sunlight, *F* is smaller than one, and decreases with increasing wavelength, however [28,29]. As the sun lowers, more direct sunlight is reflected off the water surface [28], so relatively more diffuse light is transmitted. It was therefore calculated that with lowering sun, an increasing portion of light at longer wavelengths was removed from subsurface radiation, giving underwater light a bluer colour [30]. We observed in agreement with Equation 3, that if completely diffuse sunlight could not be assumed, *R/B* considerably decreased, and *B/G* increased, as the sun lowered below 15°. For that reason we excluded all measurements taken while the sun was below 15°, if completely diffuse daylight could not be assumed.

### 2.5. Field Surveys

**Figure 2.**
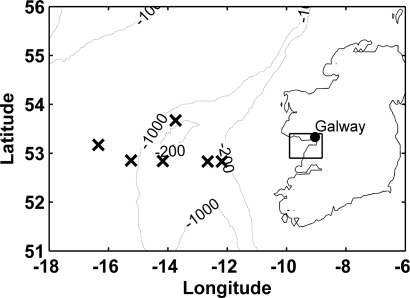
Map of Ireland and the North Atlantic, the rectangle contains the survey area in Galway Bay. The contours at 200 and 1,000 m depth indicate the Porcupine Bank, and the x symbols station locations in the Southern Rockall Trough.

Seven one-day surveys were performed in the coastal waters of Galway Bay (Figure 2) at different times of the year, during the period from August 2003 to February 2008. During all surveys, the CP885 digital camera was employed in combination with the tube breaking the water surface (Figure 1a), while during the last survey the ECOshot was simultaneously used (Figures 1c and 1d). During one Galway Bay survey, additional pictures were taken deploying the camera in the floating housing (Figure 1b). The stations were located in, and in the vicinity of, the River Corrib plume in the upper Northern half of the inner bay (Figure 2). The weather and sky conditions for the different surveys were highly variable. A cloudless sky was observed throughout the initial Galway Bay survey in August 2003 [22], while during the last two surveys in November and February, the skies were completely overcast. For the majority of surveys, in April and July, the weather was very unstable, however, and measurements were made in the presence of uncertain sunshine and varying cloud cover.

The composition of the water surface was analyzed for yellow substance absorption ah(440), chlorophyll concentration and suspended sediment concentration, SPM. The water analysis methods are described in [22], with a modification in the spectrophotometric method of chlorophyll determination. Instead of determining chlorophyll *a* only, both chlorophyll *a* and pheopigments concentrations were retrieved from absorbance measurements at 665 and 750 nm before, and after acidification [31]. Total chlorophyll was taken to be chlorophyll *a* plus pheopigments. Using total chlorophyll, instead of chlorophyll *a*, possibly affected Equation’s 2 offset, *m_c_*, but not its exponent, *n_c_* [22]. During the analysis of the last Galway Bay survey, Survey VII, over acidification of the chlorophyll extract was suspected, resulting in possible under or over estimations. We felt this to be acceptable, if the measurements were used to compare the responses of the CP885 and ECOshot digital cameras while imaging the water surface. For the first two and final survey, plume depths at the stations, as defined by the depth of the pynocline, were derived from profiles of temperature and salinity.

Supplementary pictures were taken outside Galway Bay, during a cruise on the RV Celtic Explorer in the Southern Rockall Trough in the North Atlantic (Figure 2), using the CP885 in the floating housing (Figure 1b). The survey was performed 1st–10th October 2004, so light intensities and solar altitudes were generally low. Water samples for analysis of yellow substance, chlorophyll and SPM were not obtained, as the deployment of the camera in the floating housing was an engineering test.

## 3. Results

### 3.1. Response Spectra

**Figure 3.**
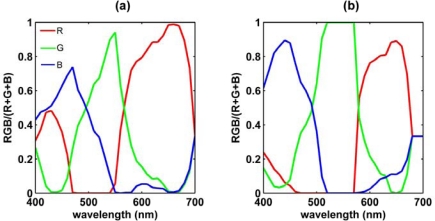
Response spectra of (a) CP885, and (b) ECOshot digital cameras. Response was defined as *RGB* values in bits, normalized to their total. Camera settings were auto exposure mode using Exp. +/− = 0 eV, and white balance ‘Direct Sunlight’.

In this study *RGB* (*I_tot_* = 400) was used, which contains colour but no intensity information. The *RGB* values measured in the spectrophotometer were therefore divided by total response, *I_tot_*, and plotted against wavelength (Figure 3).

The resultant spectra were comparable to the spectral sensitivity curves for the red, green and blue cones of a standard human observer [32]. The response spectra illustrated that both digital cameras are fundamentally three-band radiometers, with bands centred on wavelengths in the red, green and blue parts of the visible spectrum. The bands were, similar to human vision, broad and largely overlapping. A sensitivity of the red channel for blue light, and a sensitivity of the blue channel for red light, was measured for both cameras. The response of the red channel for light at the blue end of the spectrum for the CP885 (Figure 3a) was similar to the spectral sensitivity curve of human vision [32]. A spectrum measurement of the CP885 in the ‘Cloudy’ white balance setting showed an increased sensitivity of the red band for blue light, and a slightly decreased sensitivity of the blue band for red light [33]. A possible explanation for this change could be that the camera adjusts for the blue cast of downwelling light, caused by the presence of clouds and shades. Light in the green part of the spectrum showed additional response in either the red or blue channel of the CP885 (Figure 3a), similar to the response of red and blue cones of human vision [32]. The red and blue channels of the ECOshot were unresponsive to green light between 520 and 570 nm, and as a consequence *G/I_tot_* equaled one for these wavelengths (Figure 3b). This was perhaps related to the ECOshot’s design as an underwater camera, considering that underwater light is predominantly blue-green to green in oceanic and coastal waters [34]. The centre wavelengths of the red and green bands were similar for both cameras. Compared to the ECOshot, the CP885’s blue channel was found to be closer to cyan (Figure 3).

The radiometric response of band *i* = *R*,*G*,*B* of the camera to irradiance *E* could be described by:

(4) $$i=\int_{400-700}E(\lambda )\cdot s_{i}\;(\lambda )\cdot d\lambda$$

with *s_i_(λ)* the sensitivity of band *i*, as defined by the red, green and blue response curves. As a first approximation, the digital camera could be regarded as a three narrow-band radiometer by expressing the sensitivity of band *i* using a Dirac delta (*δ*) function so that it had one value around its centre wavelength *λ_i_*, and was zero elsewhere. Applying this assumption, Equation 4 simplified to:

(5) $$i=E\left(\lambda_{i}\right)\cdot s_{i}\left(\lambda_{i}\right)=E\left(\lambda_{i}\right)\cdot s_{i}$$

Values of *λ_i_*, *s_i_(λ)* and the half band width of band *i* are presented in Table 1a,b.

**Figure 4.**
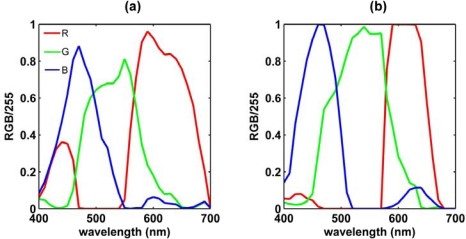
Response spectra of (a) CP885, and (b) ECOshot digital cameras. Response was defined as *RGB* values in bits, normalized to unity. Camera settings as per Figure 3.

The responsivity of a sensor is more commonly defined as the ratio between sensor output and optical power input [35]. Assuming the light source of the UV-1601 to be constant over the range of wavelengths, the *RGB* values of the separate bands were taken to be a measure of this responsivity. The *RGB* values were divided by maximum value, 255, to scale to unity. The resulting spectra (Figure 4) were slightly different from the spectra normalized to total response (Figure 3), because the denominator was a constant, and not dependent on the responses of the other bands at a particular wavelength. The centre wavelengths of the red bands of both the CP885 and the ECOshot derived from the latter were longer (Tables 1 and 2), as the denominator *R* + *G* + *B* decreased with increasing wavelength towards the red end of the spectrum (Figure 3). For the ECOshot the centre wavelength of blue band was shorter (Tables 1 and 2), as the denominator *R* + *G* + *B* also decreased towards the blue end of the spectrum (Figure 4b). At the same time, for both cameras using response values normalized to their total (Figure 3), the red and blue bands were wider, while the green bands were narrower, and the peak transmissions were more uneven (Tables 1 and 2). For the ECOShot camera, images of the projected beam in blue and red light around the center wavelengths resulted in over exposure, i.e., *R* and *B* values of 255 (Figure 4b). The peak transmission values and centre wavelengths for the blue and the red band presented in Tables 1b and 2b are therefore approximations. As a result, the peak transmission values of *R/I_tot_* and *B/I_tot_* of the ECOshot (Table 1b) are thought to be under estimations.

### 3.2. In Situ Comparison between the Digital Camera and the Ocean Colour Sensor

**Figure 5.**
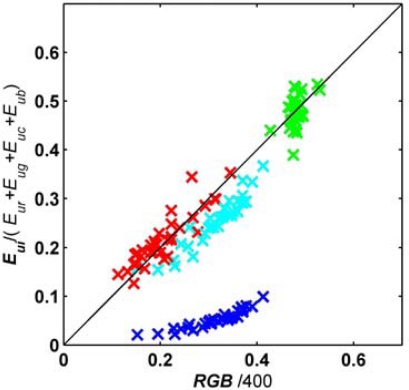
Scatter plot of OCS irradiance values in digital counts (DC), divided by total counts, against CP885’s *RGB*(*I_tot_* = 400) values, obtained *in situ* in Galway Bay. The red marks indicate OCS red and CP885 red channel, the green marks OCS green and CP885 green channel, the cyan marks OCS cyan and CP885 blue channel, and the blue marks OCS blue and CP885 blue channel. The line shows a one-to-one relation.

The aim of this paper is to assess the use of a conventional digital camera, as a more traditional radiometer, in an aquatic lighting environment. The irradiance measurements of the OCS of water leaving light, in digital counts and normalized to their total, were therefore compared with the *RGB* values of the images of the water surface captured with the CP885 (Figure 5).

The OCS measurements during the survey in November (Survey VI) had to be disregarded as light levels were too low for the OCS to accurately measure water leaving light. Figure 5 shows how well the red, green and cyan bands of the OCS correspond with the corresponding red, green and blue bands of the CP885. The response of OCS’s blue channel was too small, however. This could be explained by the centre wavelength of cameras blue band being located closer to the cyan, than the blue band of the OCC band (Table 1). Also, the photocells of the OCS are more sensitive to light at the red end, and less to light at the blue end, of the spectrum [7]. This is reflected in the increasing sensitivity of the OCS from blue, to cyan, to green to red light (Figure 5).

### 3.3. Relationships between RGB Ratios and Water Quality Parameters

*Water composition*: in Galway Bay, where the River Corrib is a major source of yellow substance, this substance was found to be the most variable optically active component, ah(440) ranging between about 0.1 and 2.8 m^−1^ over all measurements, and with survey averages ranging from 0.3 to 1.1 m^−1^. Chlorophyll concentration in the Bay varied between 0.6 and 9 mg m^−3^, with survey mean concentrations of about 2 to 3 mg m^−3^. SPM concentrations were low, an average value of 2.5 mg L^−1^ found, with a mineral fraction, MSS, of about typically 50%.

**Figure 6.**
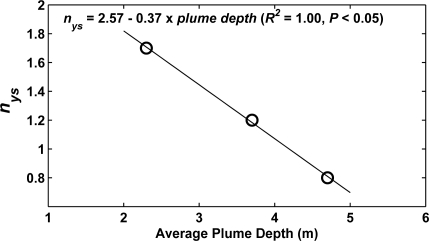
Scatter plot of slope *n_ys_* for surveys I, II and VII (Table 3a), against average plume depth during these surveys. The continuous line and the equation show the result of a reduced major axis regression between plume depth and *n_ys_*.

*Yellow substance*: highly significant linear relations between *R/B*, and yellow substance absorption were observed (Table 3a). A regression of all Galway Bay survey data resulted in a relation of:

(6) ah(440)=1.25 R/B−0.3

(*R^2^* = 0.82, *P* < 0.001), with an uncertainty in ah(440) estimation of ±0.2 m^−1^. When data of the individual surveys were regressed, regression equations varied significantly, but did not routinely result in more precise estimations (Table 3a). The variation in the regression slope, *n_ys_*, strongly related to the average plume depth within the survey area (Figure 6).

The negative linearity illustrated that the deeper the plume, the more sensitive the optical signal was to changes in surface yellow substance concentration. This correspondence could be explained by both buoyant, yellow substance rich plume water, and underlying clearer coastal water, contributing to the optical signal when the optical depth was deeper than the plume depth.

*Chlorophyll*: the power relation between *B/G* and chlorophyll concentration was less robust, but significant (Table 3b). The precision of the estimation of chlorophyll concentration improved considerably if a survey specific calibration, instead of a general calibration using all Galway Bay survey data, was used (Table 3b). The large range of exponential slope values, *n_c_*, was found to correspond with varying levels of covariance between chlorophyll and yellow substance concentration during the surveys [36]. Both chlorophyll and yellow substance absorb blue light, so the more yellow substance co varied with chlorophyll, the more sensitive *B/G* was to changes in chlorophyll concentration. A higher sensitivity was illustrated by a smaller negative exponent *n_c_*, and a higher coefficient of determination *R^2^*. Negative co variance values between yellow substance and chlorophyll corresponded with positive values of *n_c_* and small coefficients of determination. The regression slope between yellow substance and chlorophyll appeared to be inversely proportional to *n_c_*, and proportional to *R^2^* [36].

*White balancing effects*: the colour of the scene under consideration can alter *RGB* values of the imaged object, through white balancing of the digital camera. Colour as perceived by a human observer, and hence a digital camera mimicking a human observer, can be quantified by calculating hue (*H*) and saturation (*S*) values from the *RGB* values (using Matlab function rgb2hsv). The survey mean values of *H* ranged between 120 and 148 degrees (0.333 and 0.411 on a scale from 0 to 1), while *S* ranged between 0.516 and 0.616. As a test for dependence of the relation between *R/B* and ah(440) on water colour, the varying *H* and *S* values were regressed against *n_ys_* and *m_ys_* (Table 3a). The same was done for the relation between *B/G* and chlorophyll by regressing *H* and *S* against *n_c_* and *m_c_* (Table 3b). A significant relation was not retrieved, and it was therefore confirmed that using a fixed white balance settings, as during our measurements, the colour of the water surface didn’t effect the way the *RGB* ratio changed with changing ah(440) or chlorophyll concentrations.

**Figure 7.**
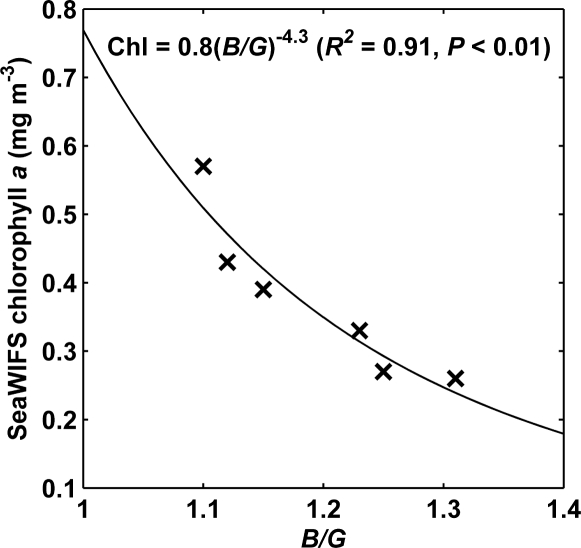
Scatter plot of chlorophyll *a* concentration derived from SeaWiFS monthly mean values [37], against *B*/*G* obtained using the *in situ* CP885 digital camera (Figure 1b) during the survey in the Southern Rockall Trough. The continuous line and the equation show the result of a reduced major axis regression between ln(*B*/*G*) and ln(Chl *a*).

*Ocean observations*: deploying the CP885 in the floating housing (Figure 1b) in Galway Bay, instead of imaging tube leaving light (Figure 1a), didn’t improve our results for ah(440), and only slightly for chlorophyll, monitoring. The camera in the floating housing was very useful, however, while on deep water surveys, when the high water line of the research vessel didn’t allow for the use of a tube. The observed colour ranges in the North Atlantic, using the floating system, were smaller than in Galway Bay, but variation was big enough to recognise biological patterns over the Porcupine Bank. According to an image of SeaWiFS monthly mean values [37], chlorophyll *a* concentrations were lower than 0.6 mg m^−3^, around the time of our survey in the Southern Rockall Trough (Figure 2). A significant power relation (*R^2^* = 0.91, *P* < 0.01) between *B/G* and chlorophyll *a*, estimated from SeaWiFS global, 9-km resolution, monthly mean of October was retrieved, implying the camera’s potential in clearer oceanic waters (Figure 7).

### 3.4. Variation in down Welling Daylight

The camera was applied under a wide range of sky and weather conditions, including extremely low light levels during the surveys in November and February (Surveys VI and VII), caused by thick cloud cover and low solar elevation angles (varying from 12° to 22° and 17° to 24° respectively). A systematic correspondence linking the different conditions to the diverse observed relations (Table 3) was not retrieved.

It was believed that the colour variation of natural daylight contributed to statistical errors, however. The average *B/R* and *G/B* values of images of subsurface down welling irradiance, using the underwater ECOshot camera, were found to be 1.06 ± 0.04 and 1.03 ± 0.03 respectively. The variations during the last survey were thought to be relatively small, as the sky was completely overcast during all our measurements. We didn’t use our OCS measurements of down welling light, because we didn’t know the accurate irradiance per count values, and suspected drift of the instrument. Previous OCS measurements of natural daylight under a wide range of sky conditions, showed ratios of down welling irradiance at any two OCS bands, measured between 10 a.m. and 4 p.m., varied within 10% of their mean values [7]. It can be derived that a variation of 10% would result in an uncertainty of ±0.1, in the determination of ah(440) from *R/B* values, and in a natural log uncertainty of ±0.1 in the estimated chlorophyll concentration from *B/G* values.

### 3.5. In Situ Comparison between the Cameras

**Figure 8.**
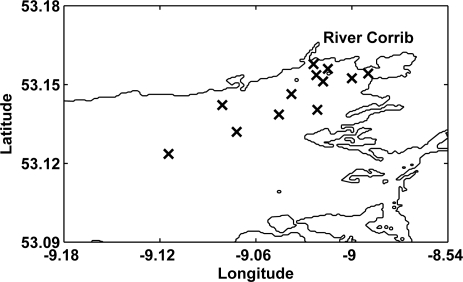
Map of upper northern half of inner Galway Bay, Ireland, (Figure 2) indicating the locations of the sample stations (x symbols) where both the CP885 and the ECOshot digital cameras were applied.

On 15 February 2008, as part of a 12-station survey in Galway Bay (Figure 8), pictures were taken using both the CP885 in combination with the tube (Figure 1a) and the underwater ECOshot camera (Figure 1c).

**Figure 9.**
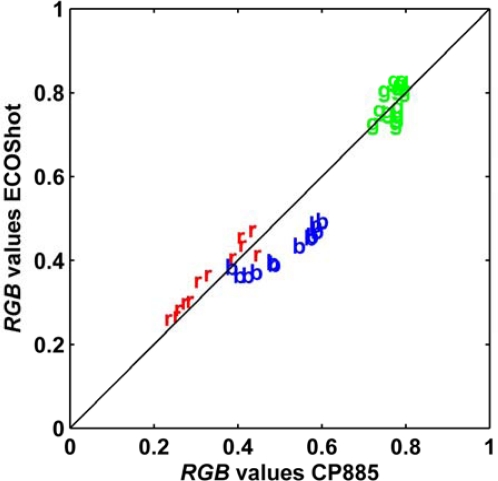
Scatter plot of *RGB* (*I_tot_* = 400) values obtained with an ECOshot against obtained with a CP885, scaled to unity, derived from images of Galway Bay surface water (*r* = red, *g* = green and *b* = blue). The line indicates a one-to-one relation.

Yellow substance absorption ah(440) varied between 0.12 and 1.68 m^−1^, while chlorophyll concentration was ranging between 0.5 and 1.7 mg m^−3^. The resulting *RGB* values of the ECOshot pictures of the water surface were almost similar to the corresponding CP885 pictures (Figure 9).

The *R* values obtained using the ECOshot were generally higher, and the *B* values lower, than those obtained using the CP885, while *G* values were more or less the same. We were interested in how the relationships between *RGB* values and water quality depended on which camera was used. The regression slope between *R/B* and yellow substance derived form pictures taken using the ECOshot:

(7) ah(440)=1.7 R/B−0.9

(*R^2^* = 0.81, *P* < 0.001) was found to be equal to the one found using the CP885:

(8) ah(440)=1.7 R/B−0.6

(*R^2^* = 0.90, *P* < 0.001). The larger negative offset (Figure 9a) was thought to be a consequence of the ECOshot’s generally higher *R*, and lower *B*, values during the survey (Figure 9). Weaker, but significant, inverse power relations between *B/G* and chlorophyll concentration were observed using both cameras. For the ECOshot the relation was described by:

(9) ln([Chl])=−4 ln(B/G)−2.6

(*R^2^* = 0.53, *P* < 0.01), showing a notably more negative exponential slope than when the CP885 was used:

(10) ln([Chl])=−2.2 ln(B/G)−1.2

(*R^2^* = 0.49, *P* < 0.02). The steeper slope of the ECOshot was consistent with the lower *B* values (Figure 9), and hence *B/G* ratios, of the *in situ* ECOshot. This made the ECOshot’s optical signal less sensitive to changes in chlorophyll concentrations than that of the CP885, presumably because its blue channel’s centre wavelength was 30 nm smaller (Tables 1a and 1b), and therefore more affected by yellow substance absorption.

## 4. Discussion

It has been shown how ‘off the shelf’ digital cameras can be used as an optical instrument to quantify water quality parameters of marine waters. Measured response spectra of the CP885 and the ECOshot illustrate that these digital cameras can be regarded as three-band radiometers. The red, green and blue bands are broad and overlapping, similar to human vision [32], but unlike more traditional narrow-band radiometers that are generally used in remote sensing of natural waters [4,38]. Despite this, known remote sensing algorithms, designed for more traditional radiometers, were successfully applied to *RGB* values of digital pictures of water leaving light to quantify water quality parameters. In addition, observed *RGB* values of the CP885 closely followed the responses in the corresponding red, green and cyan channels of the OCS spectral irradiance meter, while capturing water leaving light (Figure 5). It was concluded that the simplification of the digital camera as a three-band radiometer was a valid first approximation, when explaining the observed relationships between *RGB* values, obtained *in situ*, and water quality.

The results of an initial survey in Galway Bay, when the CP885 was used with a tube breaking the water surface attached to its lens [22], were successfully repeated in numerous subsequent surveys, under a wide range of lighting conditions. This included extremely low light levels, during the surveys in November and February, due to both low solar elevation angles and completely overcast skies. Surface yellow substance concentrations could be estimated from *R/B* values using a general calibration (Equation 6). Further research is needed to test its validity in water bodies other than Galway Bay. For the approximation of chlorophyll concentrations from *B/G* values, survey specific calibrations would be recommended as it would normally improve the precision of the estimation (Table 3b). The CP885 was deployed in a floating housing (Figure 1b) in the Southern Rockall Trough in the North Atlantic. The strong relation between obtained *B/G* values and SeaWIFS chlorophyll *a* (Figure 7) suggested that the camera could be used in clearer waters than in Galway Bay. The MSS concentrations in the sampled waters were too low, around 1 mg L^−1^, to affect the optical signal in a detectable manner. A correspondence between *R/G* and MSS, as in coastal waters off Arklow where MSS varied between 2 and 10 mg L^−1^ [20], was not retrieved. An effect of varying MSS levels on the remote sensing algorithms for yellow substance [11] and chlorophyll detection [24,39] wasn’t observed either. It would be interesting to further investigate the effect of higher MSS concentrations on digital camera observations.

The variation in the relationships between *RGB* ratios and water quality parameters (Table 3) was not proven to be a consequence of the digital camera methodology, such as changing lighting conditions or white balancing effects. If anything, the variation in the relationships between *B/G* and chlorophyll*,* and between *R/B* and yellow substance, demonstrated how sensitive the *in situ* camera was to properties of the water body, i.e. to level of covariance between chlorophyll and yellow substance [36], and plume depth (Figure 6) respectively. The latter results highlighted the possibility of the *in situ* camera’s use as a depth indicator.

The uncertainty in the estimation of yellow substance concentration using *R/B* values was about ±0.2 m^−1^. The natural log precision of the estimation of chlorophyll concentration from *B/G* values varied between ±0.3 and ±0.4, implying an error of −25% to 35% and of −33% to 50% respectively. The magnitude of this error was big, but agreed with previous estimations of chlorophyll concentration using two band algorithms [3,24,39,40]. The statistical errors in the ah(440) and chlorophyll estimations could be justified through consideration of a number of factors: variation in the colour of natural sunlight (10% for any colour ratio), inhomogeneous vertical distributions of optically active components in the water column (e.g. varying plume depth), variation in the presence of the other water quality parameters, errors in measurements of ah(440) and chlorophyll concentration, and patchiness of the water. Tube depth variation during measurements was not found to contribute to significant errors in waters as clear as in Galway Bay. It was concluded that, except for colour changes of sunlight, the uncertainties were unrelated to the actual camera deployment technique.

The *RGB* values of the *in situ* CP885 and ECOshot cameras, obtained in the surface waters of Galway Bay, were mostly similar (Figure 9). Subtle differences between the two cameras were partly explained by their different response spectra (Figure 3), in combination with the spectral distribution of the captured water leaving light. The ratio of *G* values obtained using the ECOshot, and using the CP885, was 1.04±0.04 on average. This ratio agreed with the estimated sensitivities of their green bands, *s_g_,_ECOshot_/s_g,CP885_*, being 1.05 (Equation 5, Table 1). Using Equation 5 to calculate similar relations for *in situ R* and *B* values was not successful. Despite the higher peak transmission of the ECOshot’s blue band (Figure 3), its *B* values didn’t exceed those obtained using the CP885. A possible explanation could be that the CP885’s centre wavelength of the blue band was 30 nm longer, closer to cyan, than the centre wavelength of the ECOshot’s blue band (Table 1). OCS reflectance of the water surface in Galway Bay was two to three times higher in its cyan channel (490 nm), than in its blue channel (439 nm) [41]. The higher *in situ* ECOshot’s *R* values, compared to corresponding *R* values of the CP885, could not be simply explained by the different response spectra of the two cameras, indicating that more complex colour encoding was at work. The ECOshot’s red channel was narrower and its peak transmission lower, while the centre wavelengths of the red channels of the CP885 and ECOshot were equal (Table 1). In addition, the response of the red channel to blue light, for the CP885, was not as strong in the ECOshot (Figure 3). The differences between the two cameras were minor.

The proportionality between yellow substance and *R/B* appeared to be similar for the ECOshot (Equation 7) and the CP885 (Equation 8), while the CP885 was slightly more sensitive to changes in chlorophyll concentration (Equations 9&10). It would appear that the CP885 is more effective in monitoring chlorophyll, because its blue band is closer to cyan (Figure 3; Table 1).

## 5. Conclusions

It was concluded that a conventional digital camera can act as a simple but practical three-band radiometer. Remote sensing algorithms for water quality observations, based on spectral reflectance ratios at wavelengths in the visible part of the light spectrum, were successfully applied to *RGB* values of images of the water surface. Significant relationship between *R/B* and yellow substance, and *B/G* and chlorophyll concentration were found for both ECOshot and CP885 digital cameras. Differences between the cameras could be explained by their response spectra, in combination with the colour of water leaving light. Variations in the relationships between *RGB* ratios and water quality parameters for the different surveys (Table 3) appeared to be controlled by properties of the water body, and not by the different lighting conditions or the way the digital camera encoded colour. In our experience, the auto exposure feature of the digital camera made adjusting to varying light levels very user friendly, because it was not necessary to set exposure time before measurements. Using auto exposure, both cameras performed as usual under very low light levels, even during a survey when light levels were too low to employ the OCS, set with a 10 s sample time that was suitable for other surveys.

## Figures and Tables

**Table 1.** Centre wavelength *λ_i_* (nm), peak transmission *s_i_* and half band width *HBW* (nm) of band *i*, regarding spectral response values *RGB*/(*R* + *G* + *B*) (Figure 3), of digital camera (a) CP885, and (b) ECOshot.

**(a) CP885**				**(b) ECOshot**			
	
***i***	***R/I_tot_***	***G/I_tot_***	***B/I_tot_***	***i***	***R/I_tot_***	***G/I_tot_***	***B/I_tot_***
*λ_i_*	650	550	470	*λ_i_*	650	545	440
*s_i_*	0.99	0.95	0.74	*s_i_*	0.89	1.00	0.89
*HBW*	130	80	125	*HBW*	100	90	110

**Table 2.** Centre wavelength *λ_i_* (nm), peak transmission *s*_i_ and half band width *HBW* (nm) of band *i*, regarding spectral response values of *RGB*/255 (Figure 4), of digital camera (a) CP885 and (b) ECOshot.

(a) CP885				(b) ECOshot			
*i*	*R*/255	*G*/255	*B*/255	*i*	*R*/255	*G*/255	*B*/255
*λ_i_*	590	550	470	*λ_i_*	600	540	460
*s_i_*	0.96	0.81	0.88	*s_i_*	1.00	1.00	1.00
*HBW*	110	105	70	*HBW*	75	110	75

**Table 3.** Reduced major axis regression results, using Galway Bay survey (*S*) data, for (a) ah(440) = *n_ys_* *R/B* + *m_ys_* and (b) ln(chlorophyll) = *n_c_* ln(*B/G*) + ln(*m*). *RGB* values were obtained using the CP885 in combination with the tube (Figure 1.a). Because we didn’t use *y*-on-*x* regression, the regression slopes and intercepts were slightly different from those previously presented [22]; √MSE is the unexplained error of regression.

***(a)***					
***S***	***n_ys_***	***m_ys_***	***√MSE***	***P***	***R^2^***

I	1.2	−0.2	±0.2	0.001	0.81
II	0.8	0	±0.1	0.001	0.92
IV	2.2	−0.8	±0.1	0.05	0.60
V	0.7	−0.1	±0.07	0.001	0.83
VI	1.3	−0.4	±0.3	0.001	0.83
VII	1.7	−0.6	±0.3	0.001	0.90
All	1.25	−0.3	±0.2	0.001	0.82

***(b)***					
***S***	***n_c_***	***ln(m_c_)***	***√MSE***	***P***	***R^2^***

I	−4.7	−1.0	±0.4	0.001	0.80
II	−1.6	0	±0.4	0.001	0.80
IV	3.9	2.1	±0.3	0.2	0.20
V	−4.2	−1.0	±0.3	0.001	0.66
VI	1.5	1.7	±0.3	0.05	0.37
All	−2.6	−0.4	±0.6	0.01	0.18
